# Age-Related Declines in the Ability to Modulate Common Input to Bilateral and Unilateral Plantar Flexors During Forward Postural Lean

**DOI:** 10.3389/fnhum.2018.00254

**Published:** 2018-06-25

**Authors:** Tatsunori Watanabe, Kotaro Saito, Kazuto Ishida, Shigeo Tanabe, Ippei Nojima

**Affiliations:** ^1^Department of Physical Therapy, Graduate School of Medicine, Nagoya University, Nagoya, Japan; ^2^Japan Society for the Promotion of Science, Tokyo, Japan; ^3^Faculty of Rehabilitation, School of Health Sciences, Fujita Health University, Toyoake, Japan

**Keywords:** EMG coherence, plantar flexors, common input, balance, aging, forward postural lean

## Abstract

Aging can impair an ability to lean the body forward to the edge of the base of support. Here, we investigated, using a coherence analysis, common inputs to bilateral and unilateral plantar flexor muscles to test a hypothesis that the age-related impairment would be related to strong synchronous bilateral activation and reduced cortical control of these muscles. Healthy young (*n* = 14) and elderly adults (*n* = 19), who were all right-foot dominant, performed quiet standing task and tasks that required the subjects to lean their body forward to 35 and 75% of the maximum lean distance. The electromyogram was recorded from the bilateral medial gastrocnemius (MG) and soleus (SL) muscles. We analyzed delta-band coherence, that reflects comodulation of muscle activity, between the bilateral homologous muscles (MG-MG and SL-SL pairs). The origin of this bilateral comodulation is suggested to be the subcortical system. Also, we examined beta-band coherence, that is related to the corticospinal drive, between the unilateral muscles (MG-SL pair) in the right leg. Results indicated that the bilateral delta-band coherence for the MG-MG pair was significantly smaller in the 75% forward lean than quiet standing and 35% forward lean tasks for the young adults (quiet: *p* = 0.036; 35%: *p* = 0.0011). The bilateral delta-band coherence for the SL-SL pair was significantly smaller in the 75% forward lean than 35% forward lean task for the young adults (*p* = 0.027). Furthermore, the unilateral beta-band coherence was larger in the forward lean than quiet standing task for the young adults (35%: *p* < 0.001; 75%: *p* = 0.029). Contrarily, the elderly adults did not demonstrate such changes. These findings suggest the importance of decreasing the synchronous bilateral activation and increasing the unilateral cortical control of the plantar flexor muscles for the successful forward postural lean performance, and that aging impairs this modulatory ability.

## Introduction

Functional tasks in daily activities, such as reaching out for an item, often times require shifting of the weight toward the edge of the base of support. Leaning of the body imposes higher demands on the postural control system, and age-related general declines in physical function commonly lead to difficulty in performing tasks involving weight shifting. It has been reported that elderly adults show less controlled lean path and greater sway at the maximal lean position, when compared to young adults (Blaszczyk et al., 1994). Furthermore, falls frequently occur during leaning or reaching (Nachreiner et al., 2007), and a reduced ability to lean the body forward is associated with a future fall risk (Duncan et al., 1992). Despite the widespread use of the leaning task as an assessment tool of balance dysfunction in clinical and community settings (Duncan et al., 1992; Clark and Rose, 2001), the underlying neurophysiological mechanism of the age-related impairments has not been fully elucidated yet.

Control of posture is a complicated neuromuscular mechanism requiring effective and efficient activation of postural muscles, and a number of previous studies have explored how plantar flexor muscles are coordinated during standing as the center of mass locates in front of the ankle joint (Winter, 1995). It has been reported that the bilateral soleus (SL) muscles receive greater common input during standing than voluntary contraction (Mochizuki et al., 2006, 2007). Furthermore, recent studies have demonstrated coherence of the electromyographic (EMG) signals, that quantifies common input to two motor neuron pools (Farmer et al., 1993), between bilateral homologous plantar flexor muscles in delta band (0–5 Hz) during quiet standing (Boonstra et al., 2008; Obata et al., 2014). The delta-band coherence can reflect comodulation of muscle activity (De Luca and Erim, 2002; Lowery et al., 2007), and the origin of this bilateral comodulation during standing is suggested to be the subcortical system (Mochizuki et al., 2007; Boonstra et al., 2008). In contrast to quiet standing that requires relatively little cortical control (Murnaghan et al., 2014), challenging postural tasks can induce the cortical activity (McIlroy et al., 2003). Indeed, cortical control of posture is greater during standing on a foam than rigid surface (Baudry and Duchateau, 2012) and also during unsupported than supported forward postural lean (Papegaaij et al., 2016). Moreover, increasing the cortical contribution to postural control by voluntarily swaying the body forward and backward can result in a reduction in the comodulation of bilateral plantar flexor muscles (Mochizuki et al., 2007). It can be, therefore, expected that increasing the cortical contribution by leaning the body forward would similarly decrease the bilateral comodulation. Although it is well-recognized that elderly adults have impairments in reducing the synchronous bilateral activation of limb muscles (Przybyla et al., 2011; Lin et al., 2014), how aging impacts a way in which bilateral plantar flexor muscles are coordinated during the forward postural lean has not been examined previously.

Accordingly, the purpose of the present study was to investigate the effect of aging on the modulation of common input to the bilateral and unilateral plantar flexor muscles when changing the postural position between quiet standing and forward leaning, using the coherence analysis. More specifically, we examined the previously identified delta-band coherence between the bilateral homologous plantar flexor muscles (bilateral coherence) (Boonstra et al., 2008; Obata et al., 2014), and also assessed the beta-band (15–35 Hz) coherence within the unilateral plantar flexor muscles (unilateral coherence), as the beta-band coherence has been demonstrated to reflect the corticospinal drive to the contracting muscles (Conway et al., 1995). We hypothesized the bilateral delta-band coherence to be smaller and the unilateral beta-band coherence to be larger during the forward postural lean than quiet standing, and that the modulation would be smaller in the elderly than young adults.

## Materials and Methods

### Subjects

Fourteen young [six females, mean age ± standard deviation (SD) = 22.6 ± 0.9] and nineteen elderly adults (eight females, mean age ± SD = 70.1 ± 3.3) participated in the present study. We recruited the young adults from Nagoya University and the elderly adults from the local community. None of the subjects had neurological, orthopedic, cognitive, or psychiatric problems that influence the postural balance. They also had normal or corrected-to-normal vision, and were all right-foot dominant, which was determined by asking the predominant foot used for kicking a ball. The ethics committee of Nagoya University approved this study, and all subjects gave written informed consent before participating the experiment. The experiment was conducted in accordance with the Declaration of Helsinki.

### Tasks

**FIGURE 1:**
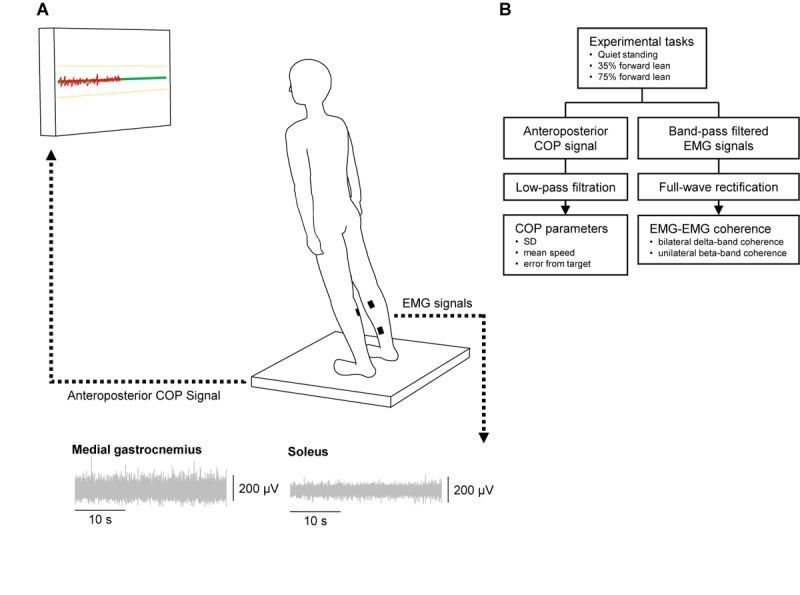
Illustration of the experimental setup **(A)** and flow chart of the experiment **(B)**. In forward lean tasks the subject stood on a force plate and was instructed to lean the body forward by dorsiflexing the ankle joint and keep the center of pressure (COP: red line) on the target line (green line) as accurately and consistently as possible. We recorded electromyograms (EMGs) from the bilateral medial gastrocnemius and soleus muscles using wireless sensors.

**Figure 1** depicts an illustration of the experimental setup and a flow chart of this experiment. There were three tasks to be performed for each subject: quiet standing and two tasks requiring the subjects to lean their body forward to 35 and 75% of the maximum lean distance (35 and 75% forward lean tasks). Before beginning the tasks, we assessed the maximum forward lean distance. The subjects were asked to stand on a force plate with their feet parallel to each other (heel-to-heel distance of 15 cm), and the feet positions were marked and kept constant during the whole experiment. They were further instructed to lean their body forward by dorsiflexing the ankle joints while maintaining the rest of their body straight. The greatest lean distance, that was determined based on the center of pressure (COP) displacement, out of three trials was adopted as the maximum lean distance and used to calculate a distance to lean in the 35 and 75% forward lean tasks. In the quiet standing task, the subjects were asked to stand quietly and look at a fixation sign on a PC monitor set 1 m in front of them. In the 35 and 75% forward lean tasks, the subjects were instructed to lean as when the maximum lean distance was determined. A green horizontal target line representing a 35 or 75% of the maximum forward lean and two yellow horizontal lines at +5% and -5% of the target line were displayed on the PC monitor. The subject’s COP position was also presented on the PC monitor as a red line progressing from left to right, which moved upward/downward as the subject leaned forward/backward. The subjects were asked to keep their COP on the green target line as accurately and consistently as possible. The task duration was 40 s, and the order of the tasks was randomized among the subjects. Two practice trials were performed before each forward lean task to familiarize the subjects with the tasks.

### Data Acquisition

Wireless EMG sensors (Trigno EMG sensors, DELSYS, Boston, MA, United States) were placed on the bilateral medial gastrocnemius (MG) and SL muscles according to the SENIAM recommendations^1^, after the skin was gently abrased and cleaned with alcohol, as these two muscles are mainly involved in postural control (Masani et al., 2003; Heroux et al., 2014). The sensors were placed as far as anatomically possible from each other to minimize the potential risk of cross-talk between the EMG recordings (Hansen et al., 2005). EMG signals were amplified and filtered (band pass filter of 20–450 Hz) using a bio-amplifier (Trigno Wireless System, DELSYS, Boston, MA, United States), and sampled at 2000 Hz. Force signals were also recorded using a force plate (Tec Gihan, Kyoto, Japan) at a sampling rate of 1000 Hz to compute the COP position. A customized LabVIEW program (National Instruments, Austin, TX, United States) was used to display the horizontal lines and COP position on the PC monitor.

### Data Analysis

**FIGURE 2:**
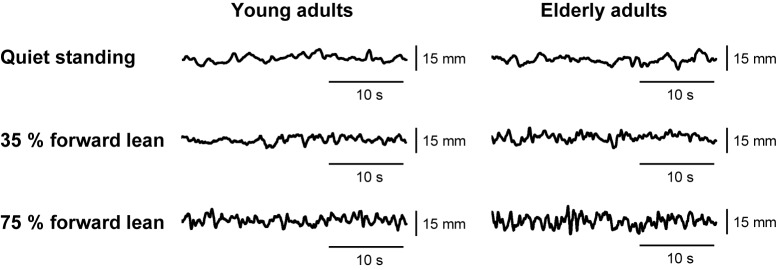
Example data of anteroposterior center of pressure (COP) displacement from an individual young adult and an individual elderly adult.

Examples of the anteroposterior COP displacement data are shown in **Figure 2**. The data during the middle 30 s of the collection period were analyzed using a customized Matlab script (MathWorks, Natick, MA, United States). After low-pass filtering the anteroposterior COP signals at 15 Hz with a fourth-order zero phase lag Butterworth filter, we calculated the SD and mean speed of anteroposterior COP displacement. The SD reflects the variability of postural displacement. For the forward lean tasks, we additionally calculated the average of absolute difference between the COP position and the target line to quantify the error from the target.

Coherence between the EMG recordings was computed based on methods provided by Halliday and colleagues (Halliday et al., 1995) to quantify common input to the plantar flexor muscles. Reproducibility of coherence analysis was investigated in several studies (Pohja et al., 2005; van Asseldonk et al., 2014), and the analysis was found to be quite reliable; however, large changes are needed to demonstrate a real difference (van Asseldonk et al., 2014). We initially rectified the EMG signals, because rectification of EMG signals can increase the information about temporal firing pattern of motor unit pools (Halliday et al., 1995; Myers et al., 2003; Negro et al., 2015). We them computed the coherence function using the following equation.

$$\left|{\text{C}}_{\text{xy}}{\left(\text{f}\right)}^{2}\right|=\frac{{\left|{\text{P}}_{\text{xy}}\left(\text{f}\right)\right|}^{2}}{{\text{P}}_{\text{xx}}(\text{f}){\text{P}}_{\text{yy}}(\text{f})}$$

*P*_xx_ *(f)* and *P*_yy_ *(f)* are the auto-spectra of the signals x and y, and *P*_xy_ *(f)* is the cross-spectra at the frequency *f*. They were calculated with a discrete Fourier transform of non-overlapping segments of 1024 data points. The coherence function is a number ranging from zero to one: zero indicates that two signals are completely independent, and one indicates that two signals are identical. For each analysis, 95% confidence limit was applied to identify the significant coherence. In the present study, the coherence was estimated in the following muscle pairs: right MG and left MG (MG-MG), right SL and left SL (SL-SL), and right MG and right SL (MG-SL). We analyzed the unilateral coherence in the dominant right leg because it was expected to be used more for the control of forward lean posture than the non-dominant left leg.

We also calculated the pooled coherence function to summarize and visualize the average difference among tasks and age groups using the following equation (Halliday et al., 1995; Amjad et al., 1997).

$${\text{C}}_{\text{pooled}}\text{=}{\left|\frac{\sum_{\text{i=1}}^{\text{k}}{\text{L}}_{\text{i}}{\text{C}}_{\text{xy}}^{\text{i}}(\text{f})}{\sum_{\text{i}}^{\text{k}}=1\;{\text{L}}_{\text{i}}}\right|}^{2}$$

${\text{C}}_{\text{xy}}^{\text{i}}$ is the coherence at the frequency *f* from individual subject, *L*_i_ is the number of segments, and *k* is the number of subjects.

### Statistical Analysis

The effects of task and age on the COP parameters (SD and mean speed of COP displacement and error from the target) were assessed with a two-way (task × age) repeated measures analysis of variance (ANOVA). Similar to previous studies (Boonstra et al., 2008; Obata et al., 2014; Laine and Valero-Cuevas, 2017; Watanabe et al., 2018), we averaged z-transformed coherence over the frequency ranges of 0–5 Hz (delta) for the bilateral coherence and 15–35 Hz (beta) for the unilateral coherence, to quantitatively compare the coherence among the tasks and age group. The effects of task and age on each of the coherence values were assessed with a two-way (task × age) repeated measure ANOVA. A Greenhouse-Geisser correction was applied for sphericity, and *post hoc* analysis and planned comparison analysis between the tasks within each group were performed with Bonferroni’s correction. We additionally analyzed correlation between either the SD or mean speed of COP displacement and each of the averaged coherence values using the Pearson’s correlation coefficients. The statistical analyses were conducted using R (R Development Core Team, Vienna, Austria) at the significant level of 0.05.

## Results

### COP Parameters

**FIGURE 3:**
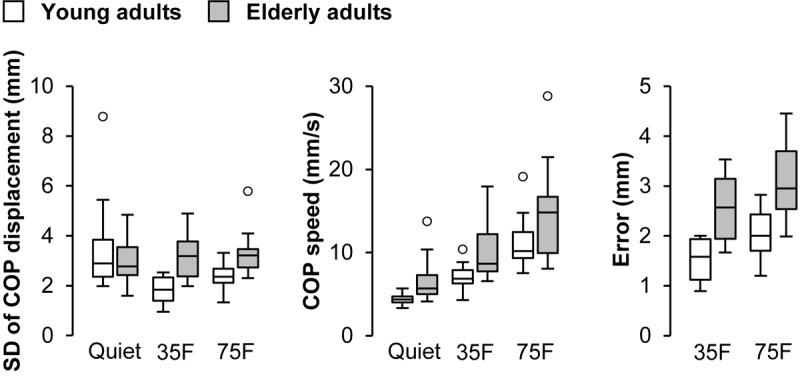
Effects of task and age on COP parameters. Data for standard deviation (SD) of center of pressure (COP) displacement, mean COP speed, and error from target line were separately presented from left to right. Tasks were quiet standing (Quiet), 35% forward lean (35F), and 75% forward lean (75F). Small circles indicate outliners.

Results of the COP parameters are presented in **Figure 3**. A two-way repeated measure ANOVA on the SD of COP displacement revealed main effects of task (*F*_1.2,38.4_ = 5.60, *p* = 0.017, η^2^ = 0.094) and age (*F*_1,31_ = 6.0, *p* = 0.020, η^2^ = 0.077) as well as their interaction (*F*_1.2,38.4_ = 8.1, *p* = 0.0044, η^2^ = 0.13). *Post hoc* analysis indicated that the SD was significantly smaller in the 35% (*p* = 0.039, Cohen’s *d* = 0.77) and 75% (*p* = 0.0012, Cohen’s *d* = 0.52) forward lean tasks than quiet standing task for the young adults. There were significant differences between age groups in the 35% (*p* < 0.001, Cohen’s *d* = 1.80) and 75% (*p* = 0.0024, Cohen’s *d* = 1.16) forward lean tasks. An analysis on the mean speed of anteroposterior COP displacement demonstrated main effects of task (*F*_1.2,37.1_ = 76.5, *p* < 0.001, η^2^ = 0.47) and age (*F*_1,31_ = 9.3, *p* = 0.0047, η^2^ = 0.16). *Post hoc* analysis showed that the mean speed was significantly higher in the 75% forward lean task than 35% forward lean and quiet standing tasks for both groups (*p* < 0.001, Cohen’s *d* = 1.41 to 2.34). Also, it was significantly higher in the 35% forward lean than quiet standing task for both groups (*p* < 0.001, Cohen’s *d* = 1.92 for young and 1.17 for elderly). An analysis on the error from the target line revealed main effects of task (*F*_1,31_ = 28.7, *p* < 0.001, η^2^ = 0.15) and age (*F*_1,31_ = 28.8, *p* < 0.001, η^2^ = 0.43).

### Coherence

**FIGURE 4:**
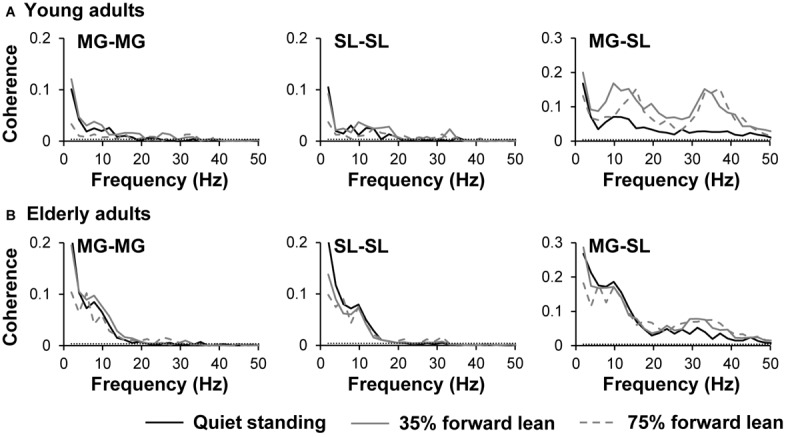
Pooled coherence spectra for young **(A)** and elderly **(B)** adults. Data are presented separately for each task and muscle pair. Horizontal dashed line indicates the 95% confidence limit. MG, medial gastrocnemius; and SL, soleus.

**FIGURE 5:**
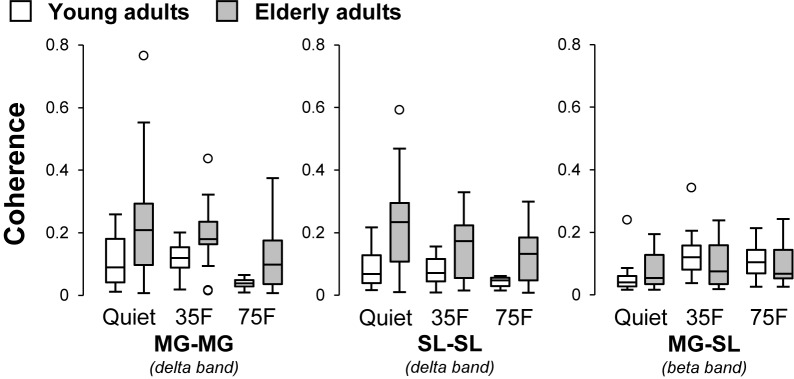
Effects of task and age on z-transformed coherence. Tasks were quiet standing (Quiet), 35% forward lean (35F), and 75% forward lean (75F). Data are presented separately for each muscle pair. Small circles indicate outliners. MG, medial gastrocnemius; and SL, soleus.

The pooled coherence spectra for the bilateral and unilateral coherences are shown in **Figure 4** to visualize the difference among tasks and age groups. The mean z-transformed delta- and beta-band coherence values are presented in **Figure 5**. A two-way repeated measure ANOVA on the delta-band coherence for the MG-MG pair revealed main effects of task (*F*_1.5,45.6_ = 6.3, *p* = 0.008, η^2^ = 0.10) and age (*F*_1,31_ = 13.4, *p* < 0.001, η^2^ = 0.15). Planned comparison analysis demonstrated that the delta-band coherence for the MG-MG pair was significantly smaller in the 75% forward lean task than 35% forward lean (*p* = 0.0011, Cohen’s *d* = 1.27) and quiet standing tasks (*p* = 0.036, Cohen’s *d* = 0.78) for the young adults. An analysis on the delta-band coherence for the SL-SL pair also revealed main effects of task (*F*_1.5,47.5_ = 6.3, *p* = 0.008, η^2^ = 0.067) and age (*F*_1,31_ = 11.5, *p* = 0.002, η^2^ = 0.20). Planned comparison analysis showed that the delta-band coherence for the SL-SL pair was significantly smaller in the 75% forward lean than 35% forward lean task (*p* = 0.027, Cohen’s *d* = 0.82) for the young adults. A two-way repeated measure ANOVA on the beta-band coherence for the MG-SL pair indicated a main effect of task (*F*_1.7,51.9_ = 17.1, *p* < 0.001, η^2^ = 0.092) and an interaction between task and age (*F*_1.7,51.9_ = 4.9, *p* = 0.016, η^2^ = 0.028). *Post hoc* analysis demonstrated that the beta-band coherence for the MG-SL pair was larger in the 35 and 75% forward lean than quiet standing task (35%: *p* < 0.001, Cohen’s *d* = 2.08; 75%: *p* = 0.029, Cohen’s *d* = 0.81) for the young adults. There was no statistically significant difference between the tasks for the elderly adults. Thus, we performed *post hoc* power analysis for detecting difference between the quiet standing and 75% forward lean task in the elderly adults using GPower (Faul and Erdfelder, 1992) and found *post hoc* power estimates of 0.55 for the delta-band coherence for the MG-MG pair, 0.66 for the delta-band coherence for the SL-SL pair, and 0.61 for the beta-band coherence for the MG-SL pair.

There was positive correlation between the SD of COP displacement and the delta-band coherence for the SL-SL pair (*r* = 0.56, *p* = 0.038) in the 75% forward lean task for the young adults. For the elderly adults, the SD and speed of COP displacement were both positively correlated with the delta-band coherence for the SL-SL pair in the quiet standing task (SD: *r* = 0.51, *p* = 0.027; speed: *r* = 0.51, *p* = 0.025). The speed of COP displacement was also correlated positively with the beta-band coherence for the MG-SL pair in the 35% (*r* = 0.53, *p* = 0.019) and 75% forward lean tasks (*r* = 0.54, *p* = 0.018) for the elderly adults.

## Discussion

The present study investigated, using the coherence analysis, the common input to the bilateral and unilateral plantar flexor muscles during quiet standing and forward postural lean in young and elderly adults. Main results indicated that the bilateral delta-band coherence was significantly smaller in the 75% forward lean task as compared to the other tasks for the young adults. Also, the unilateral beta-band coherence was found to be significantly larger in the 35 and 75% forward lean than quiet standing task for the young adults. Contrarily, such changes were not significant in the elderly adults. It is likely that elderly adults have difficulty in modulating the common inputs to bilateral and unilateral plantar flexor muscles when leaning the body forward.

Because corticomuscular coherence has been reported mainly in the beta band (Conway et al., 1995; Mima and Hallett, 1999), intermuscular coherence within one limb in this frequency range has been suggested to reflect the corticospinal drive (Farmer, 1998). Furthermore, it was recently demonstrated that the intermuscular beta-band coherence could reflect not merely the corticospinal drive but cortical control over synergistically working muscles (i.e., muscle coordination) (Laine and Valero-Cuevas, 2017; Reyes et al., 2017). As leaning the body forward supposedly requires higher cortical demands, it was expected that the unilateral beta-band coherence would be larger during the forward lean than quiet standing task. It appears that the young adults increased cortical control of the MG and SL muscles during the forward lean tasks, and the increase consequently resulted in a decrease in the SD of COP displacement, as greater beta-band oscillation and coherence are associated with steadier control of force output (Kilner et al., 2000; Engel and Fries, 2010). On the other hand, the modulation by the elderly adults was not significant. In a study by Papegaaij et al. (2016), they suggested that intracortical inhibitory activity could be lower during unsupported than supported forward leaning in both age groups. In addition, the thread/fear of losing balance was suggested to be an important factor modulating the cortical activity because the modulation was greater as the center of mass moved closer to the edge of the base of support (Papegaaij et al., 2016). Thus, it can be proposed from the present and previous findings that, although an increase in difficulty of postural task and the associated fear of falling can modulate the cortical activity in elderly adults, they have a reduced ability to cortically coordinate the synergistically working muscles as a functional unit (Laine and Valero-Cuevas, 2017; Reyes et al., 2017). Because aging has been reported to increase the beta-band corticomuscular and intermuscular coherences (Johnson and Shinohara, 2012; Watanabe et al., 2018), this study’s findings may be specific to tasks that involve the bilateral activation. The capability to increase the beta-band coherence may, hence, depend on the magnitude of bilateral comodulation of plantar flexor muscles, as described below.

In agreement with previous observations (Boonstra et al., 2008; Obata et al., 2014), we found the delta-band coherence between the bilateral homologous plantar flexor muscles during quiet standing, and this bilateral comodulation was greater in the elderly than young adults in all tasks. An important and interesting finding was the smaller delta-band coherence for the MG-MG and SL-SL pairs in the 75% forward lean task for the young adults. It appears that the young adults decreased the bilateral comodulation while increasing the cortical control of the plantar flexor muscles when leaning the body forward close to the edge of the base of support. The decrease in the bilateral comodulation could also indicate that the planter flexors were controlled more (or relatively) unilaterally. On the other hand, the elderly adults might have had difficulty in operating such a switch from bilateral to unilateral control, as evidenced by an age-related impairment in the ability to reduce in-phase coordination during a bilateral task (Temprado et al., 2010; Lin et al., 2014). Indeed, the bilateral coherence is larger during in-phase than antiphase coordination (Evans and Baker, 2003). Similar to when performing tasks involving upper and lower limb coordination (Fling and Seidler, 2012; Fujiyama et al., 2012), the present age-related decline in the modulatory ability might be ascribed to inhibitory dysfunction. It is also possible that their relatively larger delta-band coherence made it harder to modulate the bilateral and unilateral coherences.

Correlation analysis between the COP parameter and the strength of coherence revealed different patterns of correlation among the age groups. The positive correlation for the bilateral delta-band coherence during quiet standing in the elderly adults may indicate that the bilateral low-frequency oscillations influenced the force output variability (Negro et al., 2009) and thus postural sway. More importantly, in the 75% forward lean task, there was positive correlation between the SD of COP displacement and the bilateral delta-band coherence for the SL-SL pair in the young adults. In a recent study by de Vries et al. (2016), the bilateral coherence was reported to depend on the degree of bilateral coordination required for the task and become smaller with the slighter bilateral coordination. Furthermore, they demonstrated the larger beta-band corticomuscular coherence with the slighter bilateral coordination (de Vries et al., 2016), suggesting an inverse relationship between the bilateral and unilateral coherences. The present results including the larger beta-band coherence in the forward lean than quiet standing task, thus, likely propose that the young adults employed a strategy shifting from the synchronous bilateral activation to unilateral cortical control of plantar flexor muscles during the 75% forward lean task, in order to realize the better performance, supporting the above-mentioned argument. On the other hand, the mean COP speed was correlated positively with the unilateral beta-band coherence in the forward lean tasks for the elderly adults. As the larger beta-band coherence has been reported not to necessarily lead to better performance in elderly adults (Johnson and Shinohara, 2012; Watanabe et al., 2018), the increase in the corticospinal drive to cope with difficult tasks might have been dysfunctional. The role of the beta-band oscillation during a bilateral coordination appears to differ between young and elderly adults but should be clarified in future studies with more sophisticated experimental manipulations (de Vries et al., 2016).

Significant differences in the mean COP speed and the error from the target line between two forward lean tasks expectedly indicate that the difficulty of the task became greater as the lean distance became larger. With respect to the coherence values, the significant decrease in the delta-band coherence was observed primarily in the 75% forward lean task. In contrast, the magnitude of increase in the beta-band coherence was not different between two forward lean tasks. These two observations may imply that the modulation of cortical activity is a primary strategy employed during a slight body leaning, and the magnitude of bilateral comodulation becomes an important factor as the body moves close to the edge of the base of support. Not merely the task difficulty but the fear of falling, therefore, are likely associated with the bilateral comodultion of plantar flexor muscles.

This study has several limitations. First, this study included planned comparisons and was slightly underpowered to examine a difference in the coherence between the postural tasks in the elderly adults. Further studies with a larger sample are needed to confirm this study’s findings. Second, we did not measure the angle of the hip, knee, or ankle joint, or activity of upper-leg or back muscles that could be involved in maintaining a forward postural lean position. As aging can reduce muscle strengths and cause joint degeneration, consideration of these factors would have strengthened our discussion. Third, visual feedback of the COP position that was provided in the forward lean tasks to keep the lean distance constant might have had some influence on the present results, although its effect on postural control still remains under debate (Dault et al., 2003; Boudrahem and Rougier, 2009). How the visual feedback affects the common input to postural muscles should be confirmed in future research. Fourth, there is a possibility that the modulation of beta-band coherence would be smaller in the non-dominant than dominant leg. Finally, we estimated the corticospinal activity using the coherence analysis of surface EMG. Although there are numerous studies evaluating the corticospinal activity using this method (Halliday et al., 2003; Hansen et al., 2005; Norton and Gorassini, 2006; Barthelemy et al., 2010; Petersen et al., 2010, 2013; Nojima et al., 2018; Watanabe et al., 2018), inclusion of intramuscular single-unit and electroencephalography recordings would have enhanced the study’s conclusion.

In summary, this study demonstrates the importance of decreasing the delta-band coherence between bilateral homologous plantar flexor muscles and increasing the beta-band coherence among unilateral plantar flexor muscles when maintaining a forward postural lean position. Aging further appears to impair such a modulatory ability. High-quality forward leaning performance likely requires a smooth shift from more synchronous bilateral activation to more unilateral cortical control of plantar flexor muscles. Therefore, interventions focusing on this factor may be beneficial for improving voluntary control of forward lean posture.

## Author Contributions

TW, KI, and IN designed the study. TW, KS, and ST performed the experiments. TW analyzed the data. TW and IN interpreted results of the experiments. TW drafted the manuscript. All authors approved the final version of the manuscript.

## Conflict of Interest Statement

The authors declare that the research was conducted in the absence of any commercial or financial relationships that could be construed as a potential conflict of interest.

## Footnotes

1 http://www.seniam.org/
